# Rapid, High-Resolution Forest Structure and Terrain Mapping over Large Areas using Single Photon Lidar

**DOI:** 10.1038/srep28277

**Published:** 2016-06-22

**Authors:** Anu Swatantran, Hao Tang, Terence Barrett, Phil DeCola, Ralph Dubayah

**Affiliations:** 1 Department of Geographical Sciences, University of Maryland College Park, MD, 20742, USA; 2 Sigma Space Corporation, 4600 Forbes Blvd, Lanham-Seabrook, MD, 20706, USA

## Abstract

Single photon lidar (SPL) is an innovative technology for rapid forest structure and terrain characterization over large areas. Here, we evaluate data from an SPL instrument - the High Resolution Quantum Lidar System (HRQLS) that was used to map the entirety of Garrett County in Maryland, USA (1700 km^2^). We develop novel approaches to filter solar noise to enable the derivation of forest canopy structure and ground elevation from SPL point clouds. SPL attributes are compared with field measurements and an existing leaf-off, low-point density discrete return lidar dataset as a means of validation. We find that canopy and ground characteristics from SPL are similar to discrete return lidar despite differences in wavelength and acquisition periods but the higher point density of the SPL data provides more structural detail. Our experience suggests that automated noise removal may be challenging, particularly over high albedo surfaces and rigorous instrument calibration is required to reduce ground measurement biases to accepted mapping standards. Nonetheless, its efficiency of data collection, and its ability to produce fine-scale, three-dimensional structure over large areas quickly strongly suggests that SPL should be considered as an efficient and potentially cost-effective alternative to existing lidar systems for large area mapping.

Lidar (Light Detection and Ranging) is one of the most accurate technologies for providing bare Earth topography as well as three-dimensional forest structure measurements^1^,^2^,^3^ the latter of which is critical for assessing ecosystem characteristics such as aboveground biomass/carbon^4^,^5^,^6^ and habitat quality^7^,^8^,^9^,^10^. However, its operational use for forest monitoring can be limited by prohibitive costs and resulting lack of coverage. In general, lidar acquisitions are expensive and time consuming because of laser energy requirements, scanning limitations (low altitude, small swaths) and data handling capabilities^3^. These constraints have thus far made it impractical to monitor large areas routinely and repeatedly. Therefore, methods combining lidar-sampling protocols with readily available multi-spectral and radar imagery have been developed to achieve wall-to-wall coverage^11^,^12^. These methods reduce lidar flight time and acquisition costs but compromise on accuracy, especially at fine spatial scales^13^.

An alternative approach to wall-to-wall mapping is now available using Single Photon Lidar (SPL). SPL is an innovative technology for rapid forest structure and terrain characterization. SPL instruments detect photons reflected off the Earth’s surface more efficiently than other lidar instruments, thereby reducing energy requirements and increasing the speed of data collection^14^. Advances in single photon detectors and timing-electronics allow dense sampling in addition to increased spatial coverage. This is desirable for many applications in urban planning, natural resource management, hydrological modeling, and forest carbon monitoring. Airborne SPL can support large-scale carbon monitoring efforts, such as NASA’s Carbon Monitoring System^15^ (CMS) by mapping forested landscapes seamlessly and rapidly without compromising on resolution and accuracy. Furthermore, SPL potentially could be flown repeatedly to detect changes that occur from forest disturbance and subsequent regrowth, providing a valuable tool for monitoring of forest ecosystems. Small-scale experiments with airborne SPL have produced encouraging results^16^ but a large-scale deployment suitable for evaluating the technology relative to conventional lidar systems has been missing and is the focus of this paper.

Lidar instruments record the time taken by a laser pulse to reach a target on the Earth’s surface, typically from an airborne or space-based platform. The time of return and intensity of energy reflected back from the target is recorded and used to derive characteristics such as height, vertical distribution of intercepted surfaces and ground elevation^17^. Commonly used airborne, multi-photon lidar (MPL) instruments are either waveform digitizing or discrete return recording. In waveform lidar instruments, the entire return signal is digitized to provide a waveform describing foliage profile and sub-canopy topography^18^ (Fig. 1). In discrete return MPL, one or more returns are recorded for each pulse to obtain a three-dimensional point cloud depicting ground elevation, canopy, and other structures such as buildings^19^.

Multi-photon lidar systems utilize high energy laser beams with long pulse widths^3^,^20^ to capture energy scattered from the target (ground surface or canopy). Single photon lidar systems in contrast, transmit a shorter and lower energy pulse and detect scattered energy more efficiently (Fig. 1). This is accomplished by recording the time of flight of every photon of energy captured by the telescope per laser pulse transmitted. Furthermore, the short channel recovery (1.6 nanoseconds) enables SPL to record multiple range measurements for each laser shot whereas recovery times in other systems are longer. A combination of these features enables SPLs to acquire high density point clouds (12–30 points per sq. meter), currently up to 30 times faster than other lidar systems, operate at high altitudes, and penetrate semi-porous obscurations such as vegetation, ground fog, and thin clouds during daytime and night-time operations.

SPL instruments typically operate in the green wavelength (532 nm) as opposed to near-infrared (e.g. 1024 nm) used by conventional vegetation lidar instruments. This is because efficient single photon near-infrared detectors are not yet widely available. While the green wavelength is beneficial for bathymetry and ice-sheet mapping, it is not ideal for vegetation studies. The 532 nm wavelength is more sensitive to background solar noise and leaf-reflectance is much reduced in the visible than in the near-infrared. SPL point clouds therefore include more background solar returns than other lidar instruments when flown during daylight conditions. This requires significant post-processing and complicates canopy structure retrieval from SPL when data are acquired in bright sunlight conditions^14^.

Recent research, mostly in support of the ICESat-2^21^ mission has shown that canopy height and ground elevation can be determined with moderate accuracy from low density photon counting data after noise filtering^16^,^20^,^22^,^23^,^24^. Scheduled for deployment in 2017, ICESat-2 utilizes a SPL instrument called ATLAS (Advanced Topographic Laser Altimeter System). The ATLAS system is optimized for bright ice sheets and returns very few photons (about 1–2) over vegetation within a nominal 15 m diameter footprint. As a result, concerns have been raised about high noise and low reflectance in the green wavelengths^24^, particularly when point density is very low and canopies are very dense. Conversely, comparisons of photon-counting data from visible and infra-red wavelengths^20^ have shown that canopy height and other vertical structure measurements were similar in most cases supporting the use of green-wavelength SPL in canopy structure retrieval if the sampling density is high. High point densities are much easier to achieve from airborne systems than from space.

Here, we evaluate the efficacy of high-density SPL in large area forest structure and terrain mapping. We used an experimental, airborne SPL instrument – the High Resolution Quantum Lidar System (HRQLS)^15^ to map the entirety of Garrett County, Maryland, USA in ~12 hours of flight time in September 2013. Garrett is the western-most county in Maryland, covering an area of 1,700 km^2^. It falls within the Appalachian physiographic province and provides a wide range of terrain and environmental gradients to test the capabilities of SPL. Large-scale lidar mapping in the United States is often performed on a county-by-by county basis, in support of a variety of requirements set by U.S. local, state and federal agencies, such as the U.S. Geological Survey (USGS) and the Federal Emergency Management Agency (FEMA). The county scale thus represents a realistic test for new mapping technology. We apply novel noise filtering algorithms and process SPL data to derive attributes describing topography and vertical vegetation structure. These attributes are then compared with field measurements and an existing discrete return MPL dataset (henceforth DRL).

## Methods

### Study Area

Garrett County is largely forested (~67%) with ~21% agricultural land. The rest of the land area (~10%) is developed, comprising of low to medium density residential neighborhoods^25^. Forests are predominantly second growth (70–90 years old)^26^ and are less fragmented than in other counties. Common tree species include mixed oaks, northern hardwoods, and hemlock-pine stands. Understory densities vary and consist of species like rhododendron, striped maple, mountain laurel and dogwood^26^.

### SPL Instrument and data collection

**Figure 2:**
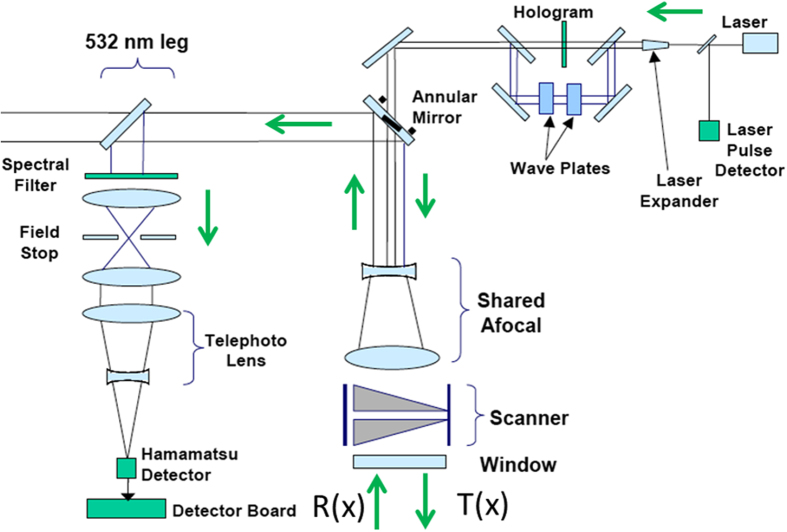
Schematic diagram of the optical component chain and flow of photons in the HRQLS instrument. Arrows show the movement of photons in the transmitted beam (Tx) and received energy (Rx).

The HRQLS instrument is designed to operate at altitudes of up to 8,534 m above ground level (Fig. 2). It consists of a single/dual-wedge scanner that collects data in conical scans with a rotating constant view angle of 20 degrees from the aircraft nadir. The Earth’s surface is illuminated by a 10 × 10 array of laser ‘beamlets’ and reflected/scattered photons are mapped to a 10 × 10 detector array. The key features that make the HRQLS system effective are its single photon sensitive detector that captures information efficiently, its use of multiple laser beamlets that increases point density and its shorter channel recovery time that enables rapid measurements. Detailed instrument specifications are available in^14^,^27^.

SPL data was collected over Garrett County in leaf-on season from a Beechcraft King Air flying at 2,286 m above ground level with an instrument swath of 1.44 km. At this nominal altitude, with a 10 × 10 array of laser beamlets, the HRQLS instrument produced 5 × 5 m target spots, resulting in ground-pixel dimensions of 0.5 m. The acquisition produced four sets of raw data: 1) laser-ranging round-trip times; 2) pointing-optics rotation phases; 3) global positioning system (GPS) airplane location measurements; and 4) inertial measurement unit (IMU) three-axis rotation data. Ranges to the measured feature point were calculated from the round-trip travel times. The pointing vector from the instrument to the measured feature point for each laser pulse was calculated from individual pointing-optics rotation phases. The instrument’s trajectory (3D path) was calculated by calibrating the raw trajectory of the airplane’s GPS location measurements with the 24-hour precise point positioning (PPP) parameters. Finally, a smoothed trajectory was calculated by combining data from the IMU with the post-processed GPS solution.

SPL point clouds were saved as LAS files^28^ that were similar to DRL data in structure and format. All files were warped into the UTM 17N projection with NAD83 horizontal datum and NAVD88 (GEOID 9) vertical coordinate reference system. The software package LAStools (http://lastools.org.) was used for processing lidar point clouds. Quick Terrain Modeler and ArcGIS software were used for visualizations and the R software was used for statistical analysis.

### Ancillary datasets

#### Discrete return lidar

An existing DRL dataset acquired over Garrett County in 2005 was processed and transformed to the same horizontal and vertical coordinate system as SPL. The dataset was collected during leaf-off season with an average density of 1pt/m^2^ and included only first-returns. While this was not the optimal dataset for validating SPL measurements, it provided a valuable three-dimensional reference for comparisons with SPL point clouds. Planimetric errors were not explicitly quantified during either the DRL or SPL data collection but we expect them to be similar since they both used standard GPS and Inertial Navigation Systems (INS).

#### National Geodetic Survey benchmarks

Ground elevation measurements from 176 National Geodetic Survey (NGS) benchmarks (http://www.ngs.noaa.gov/) were used to validate elevations from SPL and DRL data. NGS elevations ranged from 308 m to ~1000 m with a mean of 722.11 m. The NGS benchmarks had the same horizontal and vertical coordinate system as the lidar datasets and covered a variety of land cover classes. Elevation comparisons were conducted over all land cover types and not restricted to forested areas alone.

#### Field data

Heights of the 2–3 tallest trees in each of 71 variable radius field plots across Garrett County were measured during a field data collection for a NASA CMS study^15^ in summer of 2014. Tree heights ranged from 6 m to 37.7 m with a mean of 22.5 m and a standard deviation of 8.1 m. We used these field measurements for validating and comparing canopy top heights measured by SPL and DRL over forested areas. We did not compare SPL and DRL characteristics over agricultural land and other vegetated areas because of the low point density of DRL and the lack of field measurements for validation.

### SPL processing and analyses

#### Noise Removal

A multi-level filtering approach was developed to separate signal and noise returns using two basic assumptions: 1) Solar noise returns are randomly distributed in 3D space while signal returns are clustered; and 2) Solar noise returns have lower point densities than signal returns.

In the first filter, a vertical range bin of 30 m was used to extract the highest density signal comprising of topographic and canopy features. In addition, a 30 m bin above and below it was selected to ensure that all structural features were retained. The resulting dataset was a 90 m vertical subset of the raw SPL data along the topographic range. The second filter searched for signal returns in 3D space using 5 m voxels (cubic pixels). Lidar returns within a voxel were classified as noise if the total count in a cubic window surrounding it (27 voxels) was less than an empirically derived threshold (30 points in this case). A last filter, similar to the second was used, but had a smaller voxel size of 1 m and lower density threshold of 2 points. Three voxel sizes were found to be computationally efficient in filtering noise over majority of landscape features and noise distribution types. A large voxel (30 m) was effective for atmospheric noise removal. An intermediate 5 m voxel was optimum for filtering noise between objects (e.g. tree crowns). Lastly, a fine voxel size of 1 m was selected to remove isolated noise points along boundary layers. Details on the voxel-based algorithm and point density thresholds are available in^29^. This multi-level filtering process resulted in approximately 40,000 de-noised conical half-scans that were tiled into roughly 2500 1 km × 1 km subsets for further analyses.

#### Ground Elevation

Ground returns were identified from de-noised SPL data using a progressive Triangular Irregular Network (TIN) densification approach^30^ and then interpolated to generate a countywide Digital Elevation Model (DEM) at 2 m resolution. Similarly, DRL ground returns were classified and interpolated to generate a 2 m DEM using the same methods applied to the SPL dataset. Ground elevation values were extracted from the DRL and SPL DEMs over 176 NGS benchmarks and three sets of comparisons were made: (a) SPL vs DRL; (b) DRL vs. NGS; and (c) SPL vs. NGS.

#### Canopy Structure

Heights of SPL and DRL canopy points were computed relative to their respective ground DEMs. Canopy height metrics were then calculated from SPL and DRL point clouds in 30 m cells centered on 71 field plot locations. These metrics were essentially heights corresponding to every 5^th^ percentile of the point cloud distribution starting from the ground (P05) to the canopy top (P100)^31^. We compared height percentile metrics from SPL with DRL to detect similarities and differences in vertical structure. We also validated canopy top heights from SPL (P99, P100) and DRL (P100) with field height measurements. Lastly, SPL canopy returns were interpolated into a canopy height map at 1 m spatial resolution. The canopy height map had a higher resolution than the DEM because the leaf-on SPL data had more canopy returns than ground returns below dense vegetation.

## Results

### SPL Noise Filtering

**Figure 3:**
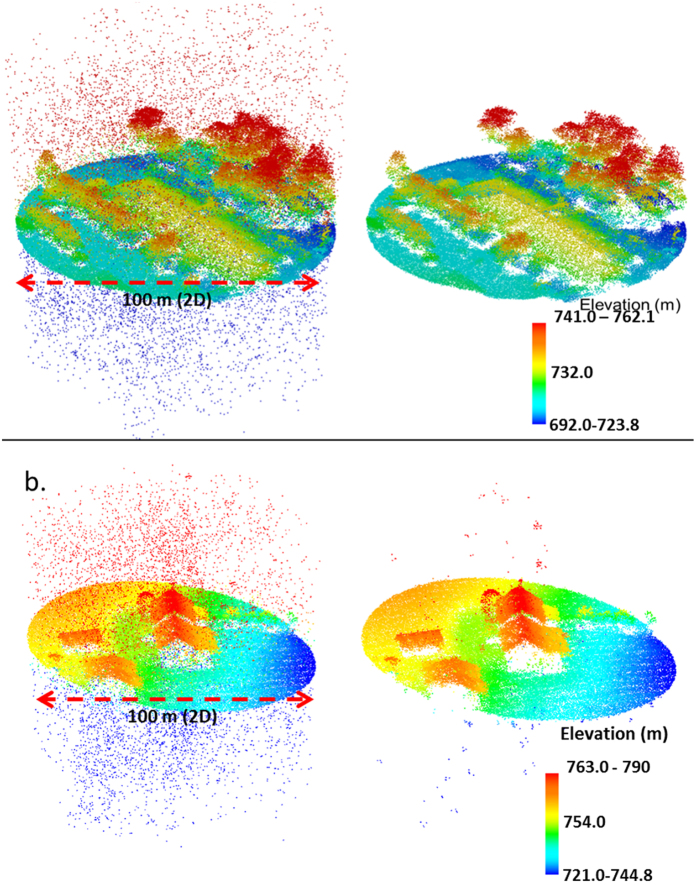
(**a**) SPL point cloud before and after multi-level noise filtering. Filtering was more effective over surfaces with low albedo (e.g. vegetation) (**b**) Noise removal was less effective over surfaces with high albedo/directional reflectance (e.g. over bright rooftops).

A quantitative accuracy assessment was not feasible because we did not have absolute references for pure signal and noise, especially within canopies and over non-forested (bright) landscapes. Nonetheless, qualitative evaluations of SPL tiles before and after de-noising showed that our algorithm was largely effective in identifying random and uniformly distributed noise points. The first filter removed ~90% of noise points above canopy and below ground (Fig. 3a). Subsequent filters functioned to eliminate majority of the noise from the boundary layers between atmosphere, canopy, and ground. A large number of residual noise points were observed around isolated targets that were further identified as highly reflective surfaces (Fig. 3b) using high-resolution imagery. In contrast, fewer residual noise points were noted over forested and agricultural lands (Fig. 3a). Clustered residual noise points were scattered throughout the dataset.

### SPL Point Cloud Characteristics

**Figure 4:**
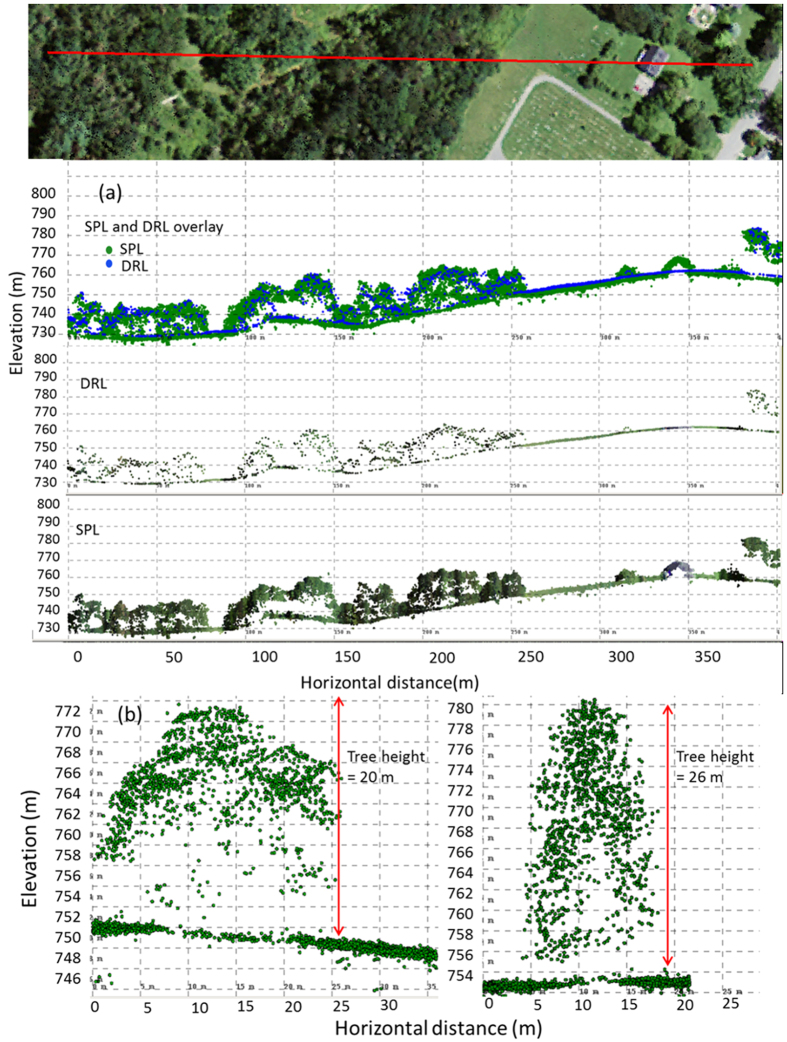
(**a**) Comparisons between Single Photon Lidar (SPL) and Discrete Return Lidar (DRL) points clouds along a 400 m transect. Note the similarities in outer canopy structure and differences in structural detail (**b**) Cross-sectional profiles of individual tree canopies from SPL data showing canopy architecture. The aerial photograph is a National Agriculture Imagery Program (NAIP) image administered by the U.S. Department of Agriculture, Farm Service Agency.

**Figure 5:**
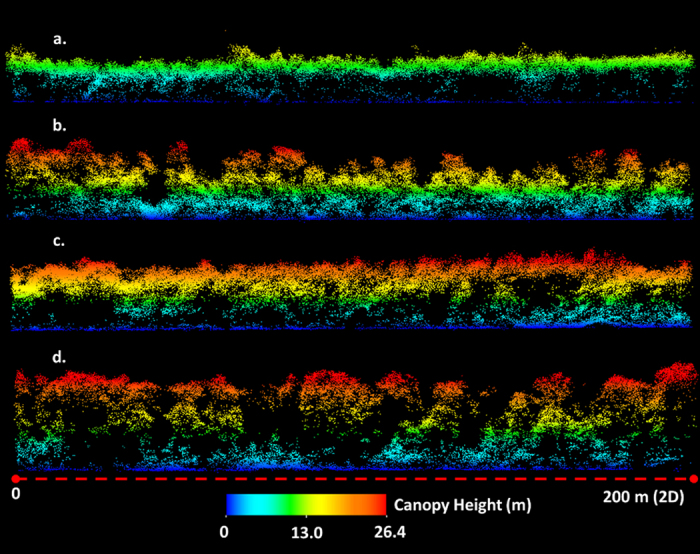
SPL point cloud profiles showing different growth patterns within a 1 km * 1 km forested area. (**a**) Short even aged stand with little understory vegetation. (**b**) Uneven aged stand composed of tall trees and dense midstory vegetation. (**c**) Even aged stand with some mid and understory growth. (**d**) Tall open stand with distinct understory vegetation.

De-noised SPL point clouds had an average density of 22 pts/m^2^ and provided detailed 3D representations of surface topography, canopy architecture and foliage distribution (Fig. 4a). Individual tree crowns and differences in spatial arrangement of canopy elements were identified (Fig. 4b). Fine-scale variations in vertical structure (e.g. overstory, and understory densities, canopy clearings, tree trunks) were also distinguished (Fig. 5).

### Ground Elevation Comparisons

**Figure 6:**
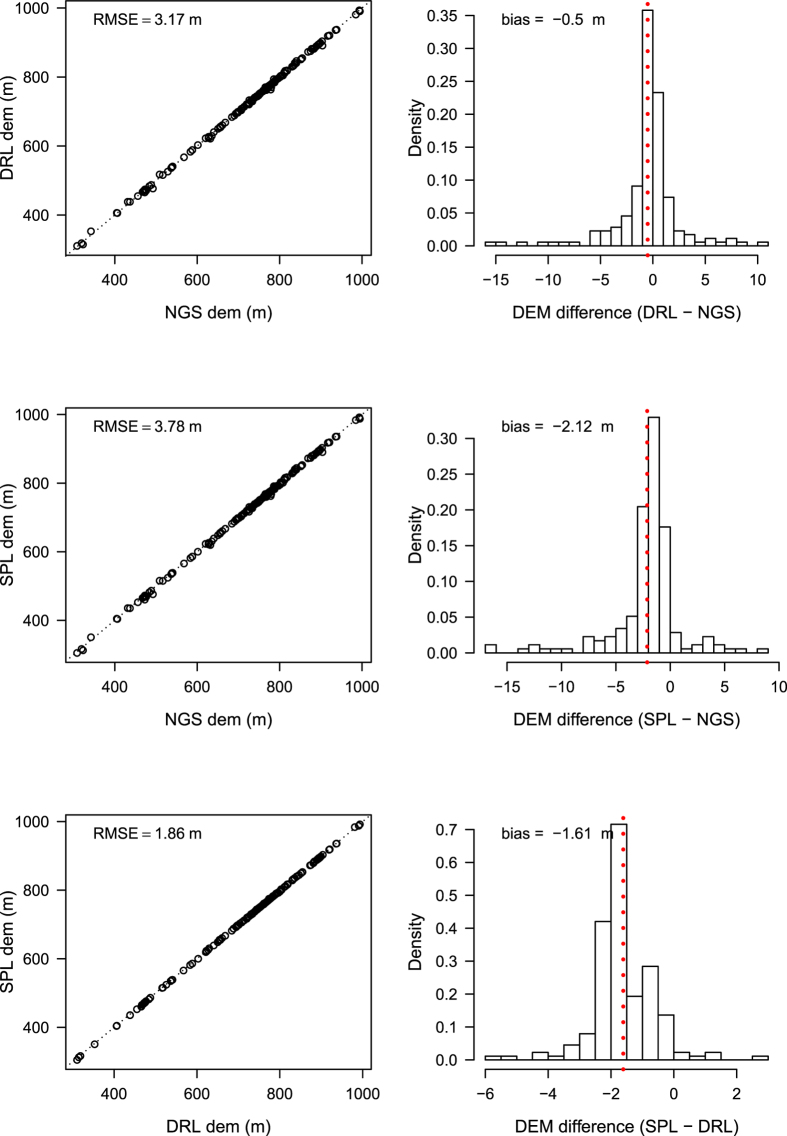
Comparisons between elevations from the National Geodetic Survey (NGS), SPL, and DRL data. The black dotted line on the scatter plot shows the 1:1 line and the red dotted line in the histogram represents mean ground elevation difference.

SPL and DRL point clouds showed distinct similarities in topographic measurements (Fig. 5a) but SPL captured more fine scale variation because of its higher point density. There was a strong correlation between NGS, SPL, and DRL ground elevations but errors were higher in the comparisons between both lidar datasets and ground survey data. SPL had an RMSE of 3.78 m (0.52% error, relative to mean NGS elevation) while DRL had an RMSE of 3.17 m (0.44% error, relative to mean NGS elevation) when compared with NGS benchmarks. SPL and DRL elevations showed a mean negative bias of −2.12 m and −0.5 m respectively when compared with NGS data (Fig. 6). SPL elevations had an RMSE of 1.86 m (0.25% error, relative to mean DRL elevation) and bias of −1.61 m when compared with DRL elevations. The biases refer to the deviations in SPL with reference to DRL or NGS. In the comparison between DRL and NGS, the bias reflects the deviation in DRL measurements with reference to NGS elevations.

### Canopy Structure Comparisons

**Figure 7:**
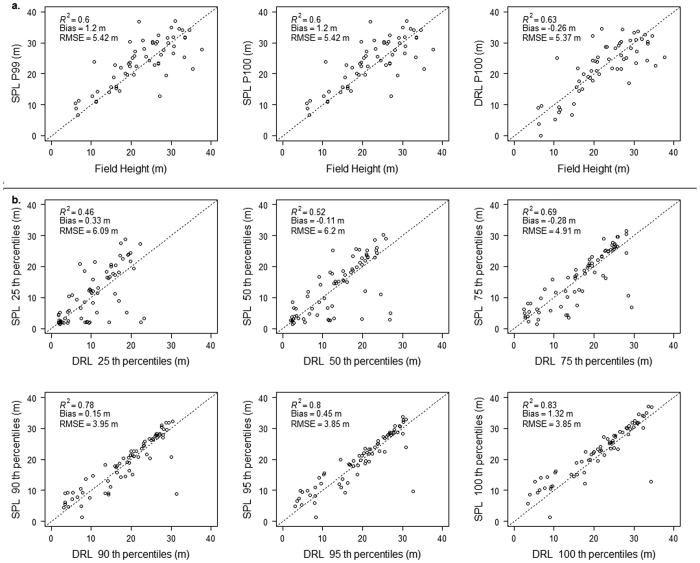
(**a**) Comparisons between Single Photon Lidar (SPL) canopy top heights (P99, P100), Discrete Return Lidar (DRL) canopy top height (P100), and maximum canopy height from field measurements. (**b**) Comparisons between canopy height percentiles from SPL and DRL data at 30 m resolution.

DRL mostly sampled the outer canopy surface given its low point density (Fig. 6a) while SPL provided more detail at the individual tree level (as would a DRL system with a higher point density). Both SPL and DRL canopy top heights showed similar accuracies (SPL_P100: R^2^ = 0.60, RMSE = 5.42 m; DRL: R^2^ = 0.63; RMSE = 5.37 m) when compared with field heights (Fig. 7a). Canopy heights from SPL had an error of 24% relative to mean field heights while DRL had a 23.8% error. SPL heights (P100) were positively biased (1.2 m) and DRL heights were negatively biased (−0.26 m) with respect to field height measurements. The P99 metric from SPL showed similar accuracies as P100 but had a lower bias (SPL_P99: R^2^ = 0.62, RMSE = 5.15 m; bias = −0.3 m). The agreement between SPL and DRL height percentile metrics (Fig. 7b) was stronger at the canopy surface with canopy top height (P100) being the most correlated (R^2^ = 0.83, RMSE = 3.85 m) but weaker within the canopy with the 25^th^ percentile height (P25) being the least correlated (R^2^ = 0.46, RMSE = 6.09 m).

## Discussion

Effective noise removal is critical for daytime SPL flying but can be challenging because of the variability in surface characteristics over large areas. Our algorithm assumed that solar noise returns were randomly distributed and had lower densities than signals but this did not hold true under all acquisition conditions. Noise densities varied with interactions between surface properties, instrument configurations, flight conditions, and sun azimuth angles in ways that were not fully anticipated or controlled in the automated de-noising process. While a detailed discussion of these interactions is beyond the scope of this study, we discuss some important factors affecting noise removal.

Filtering was least effective when there was an abrupt increase in noise density. Such increases occurred over highly reflective surfaces (e.g. such as bright rooftops solar and solar panels). Interactions between these surfaces and the sun’s azimuth angle created reflectance hotspots that resulted in much higher noise densities than expected. These points were misclassified as signal by the de-noising algorithm. Thus, there may be a need to account for surface albedo in filtering algorithms.

Filtering was also problematic when there was a clustering of noise points (as against random distribution). Clustered points were eventually traced back to a higher than expected detector ‘dead’ time in the Garrett County acquisition. The delay in recording reflected/scattered photons caused spurious clumping in the lidar point clouds. These points were not effectively filtered because our algorithm treated them as signal instead of noise. Note that these errors generally occurred over non-forested areas that comprised less than 10% of the landscape in Garrett County^25^. They did not affect our canopy structure retrievals significantly but may have increased errors in topographic maps.

Filtering was more effective over forested and agricultural lands. This was because of the relatively low albedo and anisotropic properties of vegetation that resulted in more uniform noise distribution. The signal to noise ratio was relatively consistent and densities of signal returns were higher than noise, enabling effective filtering. Clustered noise points were observed over forested areas but were distinct outliers and could potentially be filtered out from canopy height maps with further processing. Overall, our results suggest that high solar noise in SPL relative to other lidar systems may not be a limiting factor and that automated methods similar to the one developed here are appropriate for daytime acquisitions. However, more studies are required to improve filtering within canopies and at the fuzzy canopy-atmosphere boundary layers.

Lidar is a primary data source for high quality terrain mapping applications such as the National 3D Elevation Program (http://nationalmap.gov/3DEP/). Commercial acquisitions for such programs undergo extensive quality control and bias adjustments before they meet United States Geological Survey (USGS) quality standards. The Garrett County SPL dataset was an experimental collection and our goal was not to produce an elevation product meeting USGS standards. Instead, we sought to improve our understanding of the 3D attributes measured by the HRQLS instrument and the factors influencing ground elevation accuracies in order to inform future applications in topographic mapping.

Our comparisons showed that SPL, DRL, and NGS elevations were strongly correlated but both lidar datasets showed higher errors relative to the ground survey data than to each other, suggesting geolocation or other issues between lidar and field datasets. We therefore believe the DRL and SPL comparisons are likely a better indicator of the bias and errors in SPL data. The higher mean bias in SPL elevation was probably because instrument calibrations (e.g. IMU and GPS) required for topographic mapping were not rigorously controlled in this acquisition. Most of the difference in SPL vs DRL elevations was caused by bias and to a much lesser degree by incomplete de-noising over non-forested areas. Resampling the datasets to a coarser resolution may account for geolocations errors between field and lidar data and improve the overall agreement between datasets but cannot reduce the consistent bias in SPL elevations. The observed bias can be reduced with better bias control and range calibration of SPL data, as demonstrated in subsequent data collections (Supplementary Information).

**Figure 8:**
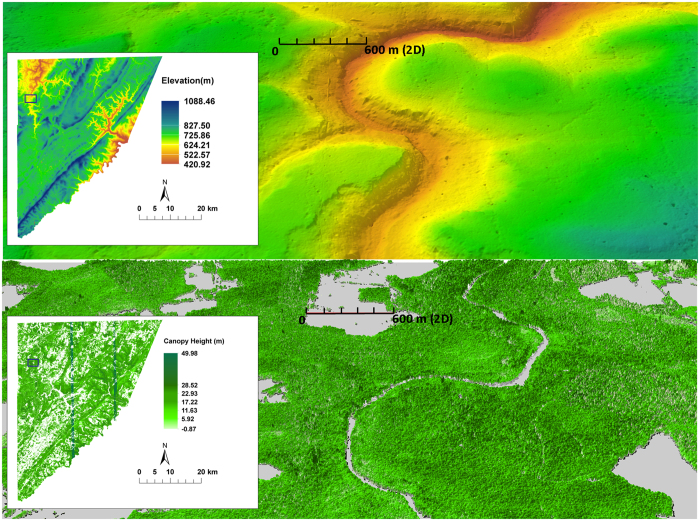
(**a**) Countywide DEM from SPL at 2 m spatial resolution. The enlarged area shows fine scale 3D topographic details along river valleys and ridges. (**b**) Countywide canopy height model at 1 m resolution. Note the spatial variability in forest structure across the landscape in the enlarged 3D map. The grey patches represent non-forested landcover classes (e.g. agriculture, water bodies, and developed land). Vertical lines in the canopy height map represent tiles with processing errors. Figure produced using ESRI software (ArcMap and ArcScene v 10.1 www.esri.com).

Although our analyses suggest the need for rigorous instrument calibration, they do not indicate a lack of precision: the precise measurements led to highly detailed imaging. This can be observed from the extensive geological, topographic, and 3-D structure in the SPL DEMs (Fig. 8a and Supplementary Fig. S1). Such detail is the hallmark of modern MPL mapping using high point densities. Consequently, most MPL acquisitions occur during leaf-off periods, to maximize the accuracy of the ground DEM (and built structures). However, achieving high point densities over large areas is expensive for MPL, as it generally requires narrow swaths, or multiple overpasses. Our experience in Garrett County suggests that SPL produces this topographic detail even during leaf-on conditions, greatly expanding the usefulness of the acquisition for multiple objectives that are normally in conflict: an accurate topography and infrastructure vs. canopy structure for forestry and carbon studies, discussed next.

An important objective of this study was to evaluate canopy structure measurements from SPL to determine its usefulness in large-scale forest mapping and carbon monitoring activities. Validations of SPL and DRL canopy top heights with field measurements showed similar accuracies, suggesting that the outer canopy surfaces observed by SPL and DRL were similar despite entirely different instrument configurations, laser wavelengths, and acquisition periods. The relatively large errors (~5 m) resulted from several sources. First, variable radius plots have no fixed diameter, so that the measured tree heights can only be associated with the center of each plot, as was measured in the field using handheld GPS (with a location error for the center usually around 3–5 m). Thus, a large tree could be at the center of the plot or 40 m away from that center, depending on its diameter. Secondly, only 1–2 heights were recorded in each plot, which is usually not enough to characterize the canopy height distribution within a plot. Lastly, tree height measurement in the field, accomplished with a laser rangefinder, is imperfect. We note that many studies, using fixed plots and careful geolocation still report lidar-derived height errors of several meters^32^,^33^.

Of interest was the higher bias in the P100 metric from SPL as against P99 when compared with field heights. This was probably the result of residual noise points at the canopy top. Similar issues are encountered with other lidar systems and are generally overcome by using the P99 metric as the canopy top height indicator^34^,^35^. However, efficient de-noising at the canopy top/air boundary interface is especially important for SPL in sunlit conditions.

Internal canopy metric comparisons (e.g. P25, P50) showed a relatively low co-efficient of determination (R^2^), high RMSE and scatter around the 1:1 line. These dissimilarities were not unexpected given the differences of year, wavelength, seasons, number of returns, and other acquisition characteristics. DRL data were first return only and were acquired in leaf-off conditions. The branching structure is usually sufficient to provide some internal detail when leaves are absent, explaining the stronger correlation at the canopy top, but the reflectivity of bark is much less in the near-infrared resulting in reduced correlations in internal structure. Some discrepancies can also be attributed to canopy growth and loss in the time interval between the two data collections. Nonetheless, the overall agreement in 3D point clouds (Fig. 4), and canopy surface measurements (Fig. 7a) is similar to findings from previous studies^20^ and provides further support to the use of green wavelength lidar in canopy height measurements if the point density is high. In particular, retrieving high-resolution canopy height and structure, over large areas on a repeated basis now appears viable (Fig. 8b). The processing speeds and the level of canopy structure detail provided by the SPL dataset may not be different from current MPL systems but the key advantage of airborne SPL is its ability to fly higher, with wider swaths (and smaller incidence angles), and collect high-density 3D measurements over large areas faster than other instruments. These characteristics are advantageous and make the system a time efficient, energy efficient and potentially cost-effective alternative for routine forest monitoring. Additionally, the impacts of disturbances, such as logging, insects, drought and fire, on forest structure, and its recovery through time could be monitored. Characterizing such canopy structure dynamics (Fig. 5) is key to our understanding of ecosystem function and habitat quality^10^,^36^,^37^,^38^,^39^.

## Conclusions

The Garrett County acquisition was the first large-area deployment of an airborne SPL instrument and a noteworthy technological development in the field of lidar remote sensing because of its speed, efficacy, and coverage. Our objective evaluations have further advanced our understanding of the strengths and limitations of the technology and the data, especially for canopy structure and terrain characterization. By comparing SPL attributes with field and DRL data, we were able to quantify biases affecting ground measurements (−1.61 m relative to DRL and −2.12 m relative to NGS elevations). This has led to further instrument and algorithm development and improved results (Supplementary Figs S1 and S2). The similarities between SPL and DRL point clouds and canopy height accuracies when compared to field data (SPL_P100: R^2^ = 0.60, RMSE = 5.42 m; DRL: R^2^ = 0.63; RMSE = 5.37 m) strengthen the applicability of green wavelength SPL in canopy structure mapping. SPL is thus poised to overcome an important limitation of current lidar systems, which is their inability to provide both wide coverage and fine, three-dimensional structure in a rapid and efficient manner.

## Additional Information

**How to cite this article**: Swatantran, A. *et al*. Rapid, High-Resolution Forest Structure and Terrain Mapping over Large Areas using Single Photon Lidar. *Sci. Rep.* **6**, 28277; doi: 10.1038/srep28277 (2016).

## Supplementary Material

Supplementary Information

## Figures and Tables

**Figure 1:**
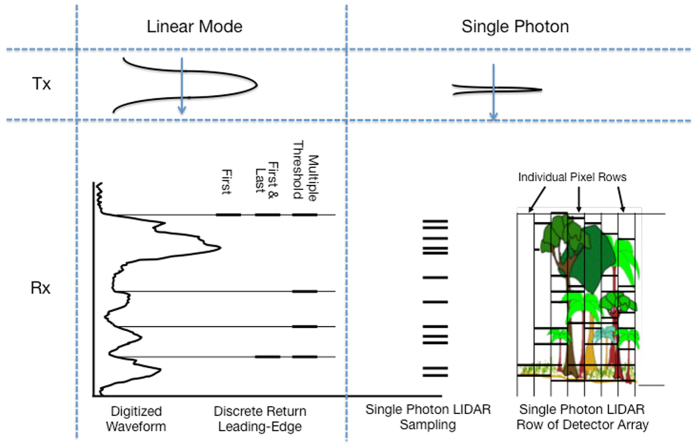
Schematic diagram showing SPL and other lidar systems. Tx is the transmitted laser pulse and Rx is the returned energy. The SPL laser pulse has a shorter pulse width than other systems. The detector consists of a 10 × 10 array that records several returns per pulse.
