# Supplementary material for: Rapid, High-Resolution Forest Structure and Terrain Mapping over Large Areas using Single Photon Lidar
Source: Sci Rep. 2016 Jun 22;6:28277. doi: 10.1038/srep28277 (PMC4916424; doi:10.1038/srep28277)
Supplement: Supplementary Information [file srep28277-s1.pdf]

Rapid, High-Resolution Forest Structure and Terrain Mapping over Large Areas using Single Photon Lidar

Anu Swatantran[1*], Hao Tang[1], Terence Barrett[2], Phil DeCola[2], & Ralph Dubayah[1]

[1] Department of Geographical Sciences, University of Maryland College Park, MD, 20742, USA

[2] Sigma Space Corporation, 4600 Forbes Blvd, Lanham-Seabrook, MD, 20706, USA

* Correspondence and requests for materials should be addressed to:

Anu Swatantran aswatan@umd.edu

Supplementary Information on Elevation Accuracies from SPL

The Garrett County single photon lidar study provided an improved understanding of the 3D attributes measured by the HRQLS instrument and the factors influencing ground elevation accuracies in order to inform future applications in topographic mapping. Collections to the USGS-3DEP specification (http://nationalmap.gov/3DEP/) allow for the adjustment of the elevation of point-cloud strips to ground-surveyed points (different points than those used to measure RMSE) which was not done in the Garrett County collection. Our evaluations suggested that accuracies could be improved with rigorous instrument calibration, careful quality control, and bias corrections. This was tested in a subsequent HRQLS acquisition over Monterey, California. In November 2013, following the Garrett County project, a 1.0km by 1.5km area containing the Naval Postgraduate School (NPS) campus in Monterey California was surveyed with the HRQLS instrument [Bathymetric Algorithm Research and Development project], with the same instrument configuration and flight parameters as used in Garrett County.

The point cloud was processed for solar noise removal and classification of the ground-return points using proprietary and commercial software. Ellipsoid elevations from a 1 m resolution DEM was then compared to 21 surveyed elevations (Figure S1 & Figure S2). Results showed an RMSE of 70.7 cm. Further analysis showed that the instrument/calibration bias (-70.1cm) constituted nearly all of the RMSE error. With the elevation bias removed, the RMSE was reduced to 0.091 m and the standard deviation was 0.093 m, which was within the 0.10 m that the USGS-3DEP program specifies for Quality Level 1 (QL1) compliance. While more work is required to improve instrument calibration and evaluate these approaches over large areas using automated algorithms, these results from a different study site provide further evidence to the utility of airborne SPL in high-resolution mapping for applications such as USGS-3DEP.

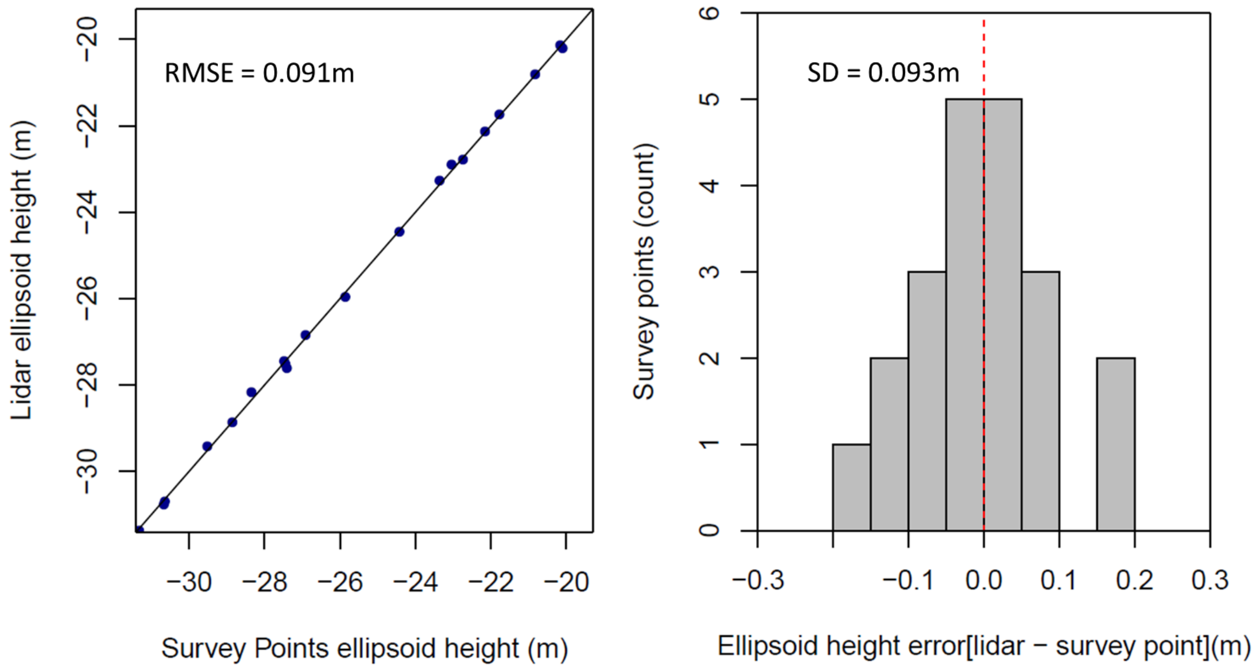

Figure S1: Vertical accuracy comparisons between ellipsoid heights from HRQLS SPL data and ground survey points over Monterey, California. Values are negative because they are not geoid-corrected. The coordinate system is UTM 10N, WGS 84.

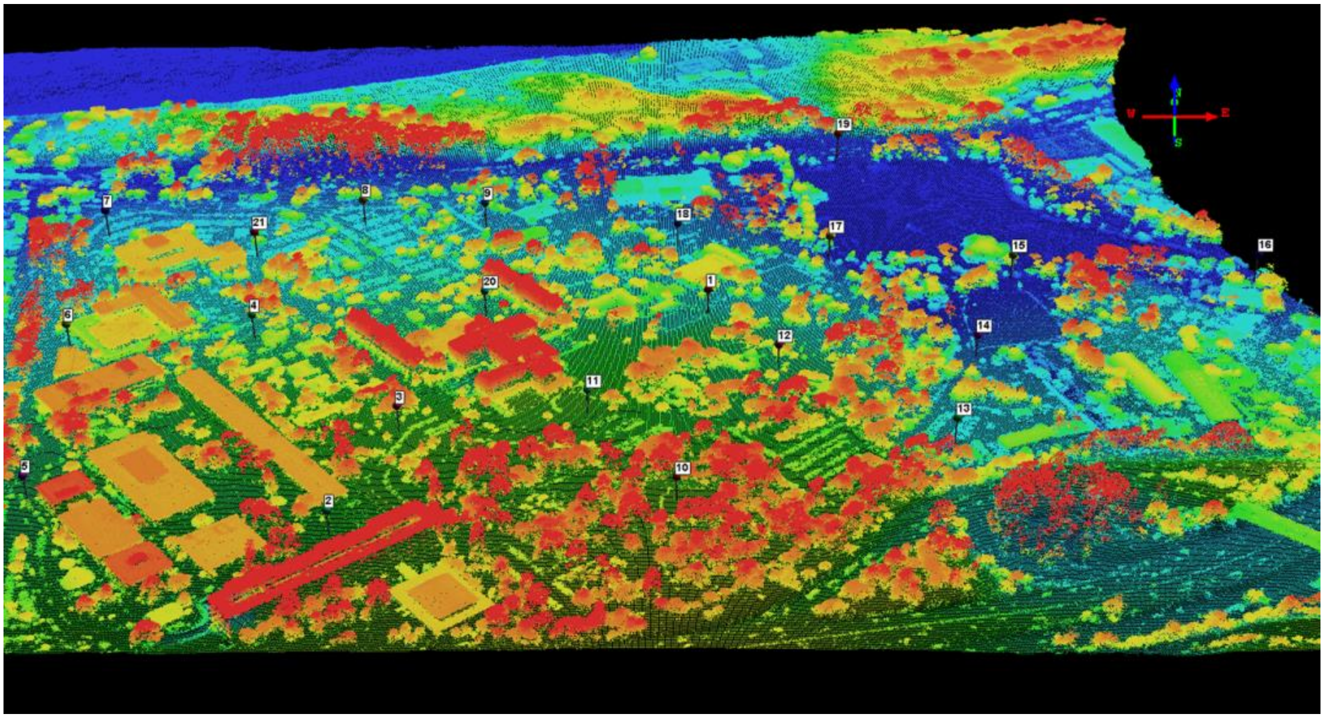

Figure S2: 3D point cloud from the Monterey, California study site showing improved noise filtering and elevation accuracies. Figure created in the Quick Terrain Modeler software v8.01 (www.appliedimagery.com)
